# COVID-19 Impacts at a Small Mid-Atlantic Liberal-Arts College with Implications for STEM Education

**Published:** 2020-11-03

**Authors:** Malcolm J. D’Souza, Katelynn Fry, Lyndsey Koyanagi, Andrew Shepherd

**Affiliations:** Wesley College STEM Undergraduate Research Center for Analytics Talent and Success, Wesley College, Delaware, USA.

**Keywords:** Wesley College, COVID-19, STEM student, Online, e-learning, Faculty

## Abstract

During the COVID-19 pandemic, with very little preparation and within a brief span of 48 hours, the Wesley College STEM faculty and students triaged into a remote-only form of instruction. Wesley College STEM student COVID-19 impact surveys showed underlying gaps in economic equity, increased family responsibilities, struggles to stay motivated, social isolation, and higher levels of psychological stress. Yet, the crisis demonstrated new ways in which technology can be harnessed and allowed STEM students to reconsider how jobs and skills should be aligned. A STEM faculty COVID-19 check-in survey and interview responses revealed a quick realization that faculty could not rely solely on Wesley’s Jenzebar learning management system (MyWesley). To engage their students and to create a supportive learning environment, STEM faculty sought new strategies and approaches for a diverse set of STEM learners. For synchronous e-teaching, the faculty used the Microsoft-Teams and the Zoom video conferencing platforms. Faculty only adopted MyWesley to execute dedicated asynchronous tasks (laboratory assignments, reports, exams). The STEM students were overwhelmingly positive about STEM faculty availability during the crisis. Still, both faculty and students indicated a much stronger preference for the face-to-face delivery of their course content via a traditional classroom setting.

## 1. Introduction

Wesley College (Wesley) is a minority-serving, primarily undergraduate institution located in Delaware’s Kent County (Wesley College, 2020). Wesley offers 28 undergraduate majors, and five, biology, biological chemistry, environmental science, environmental policy, and mathematics are in the STEM (science, technology, engineering, mathematics) disciplines (Wesley College Academic Programs, 2020). Students in biology can obtain a concentration in microbiology, and biological chemistry undergraduates have a mandatory informatics requirement (D’Souza, Barile, & Givens, 2015; D’Souza et al., 2015; Wesley College Academic Programs, 2020).

To serve widely ranging levels of student ability, Wesley instituted a progressive liberal-arts core-curriculum model where collaborative learning activities and course-embedded undergraduate research projects begin at the freshman level and are integral components for engagement and motivation (D'Souza, Kroen, Stephens, & Kashmar, 2015; D’Souza, 2012; D’Souza & Wang, 2012; D’Souza et al., 2017; D’Souza, Curran, Olsen, Nwogbaga, & Stotts, 2016; D’Souza, Dwyer, Allison, Miller, & Drohan, 2011; D’Souza et al., 2015; D’Souza, Shuman, Wentzien, & Roeske, 2018; D’Souza, Stotts, & Curran, 2014; Fry, Kroen, & Stotts, 2019; Neff & D’Souza, 2019; Wesley College Grants, 2020). Data-mining and geospatial analysis are core-level courses, and students in all majors can minor in Informatics or earn Informatics Certification (D’Souza, Alabed, Earley, Roberts, & Gerges, 2013; D’Souza et al., 2015; D’Souza et al., 2017; D’Souza et al., 2015; D’Souza, Roeske, & Neff, 2017; D’Souza et al., 2018; Wesley College Grants, 2020).

The Wesley College STEM Undergraduate Research Center for Analytics, Talent, and Success (STEM UR-CATS) program sponsors mentored course-embedded and independent (directed research) STEM undergraduate research projects (Neff & D’Souza, 2019; Wesley College Grants, 2020). Federal and State grants support all of the STEM UR-CATS endeavors (D’Souza, 2012; D’Souza, 2016; D’Souza et al., 2011; D’Souza et al., 2018; D’Souza et al., 2014; Neff & D’Souza, 2019; Wesley College Grants, 2020).

It is well-documented that Wesley mainly serves a student body that comes from historically underserved high schools and from households that have financial hardship (D'Souza et al., 2015; D’Souza, 2012; D’Souza & Wang, 2012; D’Souza et al., 2015; D’Souza et al., 2018; D’Souza et al., 2014). According to Wesley’s online Fact Book (Wesley College Fact Book, 2020), in Fall 2019, its undergraduate headcount was at 1035, and 973 students were full-time. At 39%, African Americans were the majority-minority population, and 7% of the Wesley students considered themselves to be biracial.

Over the past five years, there was a 32% decline in the total Wesley student population (Wesley College Fact Book, 2020). During the same time-period, freshman enrollment dropped considerably, adding to the financial distress on the College (Wesley College Fact Book, 2020). In July 2020, the College President and the Board of Trustees announced a signed acquisition agreement with Delaware State University, a historically black university (Wesley College is Proud to Join Delaware State University, 2020).

The COVID-19 (coronavirus disease, 2019) timeline suggests that the condition made its U.S. appearance in late January or early February 2020 (Centers for Disease Control and Prevention COVID-19 Response Team, 2020). In March, when enormous documented Northeast regional increases in weekly hospitalization rates and mortality attributed to COVID-19 were made public, many New England and Mid-Atlantic colleges and universities began the abrupt closing of their college campuses (Passy & Keshner, 2020). They emptied their dorms to transition to remote e-learning modes quickly (Passy & Keshner, 2020).

In step with the State of Delaware Governor’s Emergency Declaration (State of Delaware-Delaware Emergency Management Agency, 2020) and like its Northeast Corridor higher educational sister institutions, Wesley also pivoted to *remote-only* e-learning (in mid-March). In May/June 2020, as a result of the pandemic, Wesley students could apply for monetary aid through the Coronavirus Aid, Relief and Economic Security Act (CARES) that provided emergency relief funding for students who needed financial assistance (U.S. Department of Education, 2020).

Since the COVID-19 outbreak beginnings, from March to mid-August, the Wesley President, in his periodic COVID-updates to the College, reported zero COVID-19 cases on campus (Clark, 2020), and Kent County-Delaware consistently reported relatively low COVID-19 case counts (Delaware Gov, 2020). The administration, driven by Wesley’s mission to serve its students, and having instituted *Protect Wesley* (Wesley College., 2020) protocols and policies, decided on a face-to-face Fall 2020 residential experience (Clark, 2020; Wesley College, 2020). To better maintain its success with COVID containment and to control potential community transmissions, the College campus is closed to the non-Wesley community (Clark, 2020). Before entering the campus, a person must complete a daily COVID screening questionnaire (Wesley College, 2020). Additionally, periodic Wesley President COVID-updates serve as a continuous reminder for monitoring Wesley College student and staff behavior (Clark, 2020; Wesley College, 2020).

In the ever-changing COVID-19 context, this project, through a series of surveys and online interviews, provides evolving perspectives of some of the disruption and intervention phases that occurred within the Wesley STEM (student and faculty) body. In August 2020, a Delaware-based STEM-focused news organization interviewed the three student co-authors. It published a full-length article (Quinn, 2020) about the purpose of this study with some resultant outcomes. The online article provided visibility for the Wesley STEM student work while also providing information on civic and public importance to help Delaware’s higher education community navigate the COVID crisis.

## 2. Methods

In mid-May, the Wesley College Provost, in consultation with the Wesley College IRB (Institutional Review Board) Chair, authorized this project and instructed the Wesley College Registrar/Director of Student Records and the Wesley College Institutional Research team (within the Wesley IT Department) to release all pertinent, reliable, relevant, and quality STEM student data. As a result, we received the completed records (student name, gender, ethnicity, hours enrolled, class standing, email address, telephone information, social media contact information, name of major, and name of academic advisor) for 68 STEM majors. In an attempt to increase familiarity and gain more survey responses, an initial email was sent to all 68 STEM majors introducing the COVID-19 student research team.

Owing to observational difficulties of the COVID-19 pandemic’s longstanding disparities in student life decisions, student challenges, and student wellbeing, a series of six surveys (see Appendix l) was created using Google Forms. The surveys were sent to each of the 68 Wesley College STEM majors through email, text messaging, FaceTime, and social media platforms (Instagram and Snap Chat). For efficiency and to gauge the effectiveness of responses, each survey was concise and was explicitly designed to gain a deeper understanding of the students’ opinions.

In contrast, 23 Wesley STEM Faculty (full-time and adjunct) were given a single survey as they provided detailed responses and more insightful answers. Additionally, student researchers interviewed (one-on-one) five STEM faculty members using available video conferencing systems (Zoom or Microsoft Teams). Appendix 2 lists the faculty survey and interview questions.

All (six) student surveys had response rates equal to or above 60%, with the average response rate being 64%. On the other hand, 83% of STEM full-time and adjunct faculty responded to every item of the faculty survey.

Google Forms, Google Sheets, and Microsoft Excel were utilized for data collection, organization, and analysis. After the data was cleaned, the design platform Canva was used to create a series of infographics for public view. The COVID-19 project student researchers presented their outcomes as two posters at the August 13, 2020, University of Delaware, Virtual Undergraduate Symposium.

## 3. Results and Discussion

During the sudden and unprecedented COVID-19 campus shutdown, Wesley making real-time decisions in the best interest of its staff and students, transitioned to *remote-only* e-learning modes using Microsoft-Teams, Zoom, and MyWesley (a Jenzabar Internet Campus Solution). In response, the Wesley IT department, in a rapid-fire process and consulting with Wesley’s STEM faculty, provided laptops, Wi-Fi hotspots, and workstations purchased through the UR-CATS program (Neff & D’Sonza, 2019) to STEM majors and faculty lacking technology (at home).

### 3.1. Tracking of the Student Survey Responses of What College Life is Like during COVID-19

Each student survey Appendix 1 captured insightful feedback on Wesley’s STEM student perceptions and opinions of the COVID-19 crisis atmosphere. Detailed below are the survey results for their technology access and use, monthly household and personal income, residential community experiences, career aspirations and goals, education trajectory, and mental wellbeing.

#### Survey #1: *Positive and negative implications of student’s access to technology at home*

**Figure-1.**
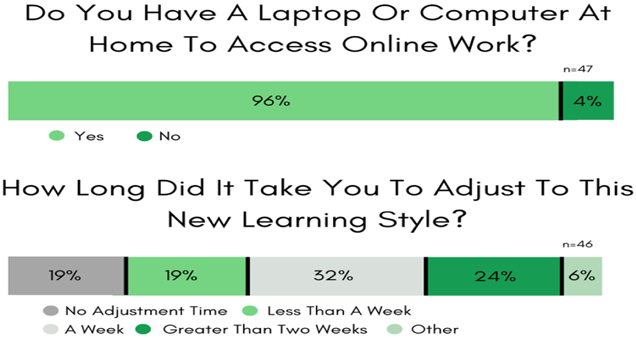
Survey #1 Results. Student’s technology access and ease of online learning modes. **Source**: Tabulated responses to Survey #1 questions, listed in Appendix 1.

Forty-seven Wesley College STEM majors responded (69% response rate) to most of the questions in this survey. As shown in Figure 1, during Delaware’s lockdown (State of Delaware, 2020), 96% of the students had access to a computational device and reliable Wi-Fi access. For lectures, depending on the STEM or non-STEM college course-level and the course instructor, students mostly used one of three remote-learning platforms, Microsoft-Teams (37%), Zoom (27%), and MyWesley (19%).

During this time of remote learning, eleven STEM majors (24%) stated that they had considerable difficulty (>2 weeks) navigating the nebulous synchronous and asynchronous e-learning formats and the chosen course platform interfaces. Also, females (15%) indicated more significant challenges with this forced immersion with technology-enabled forms of learning, but there were no discernible differences observed by race. Even though 75% of the Wesley STEM student survey respondents had access to a home printer/scanner, 21% indicated that a lack of access hurt their course participation and their course grades.

#### Survey #2: *Evaluating the effects of COVID-19’s financial impact on students.*

Survey #2 data indicated that the imposed lockdown (State of Delaware-Delaware Emergency Management Agency, 2020) led to abrupt falls in students’ monthly (family) household and personal income. There were 46 Wesley STEM students who answered (68% response rate) questions about their monthly household finances. Forty-four percent lived in a household that was impacted by pandemic-related job losses.

**Figure-2.**
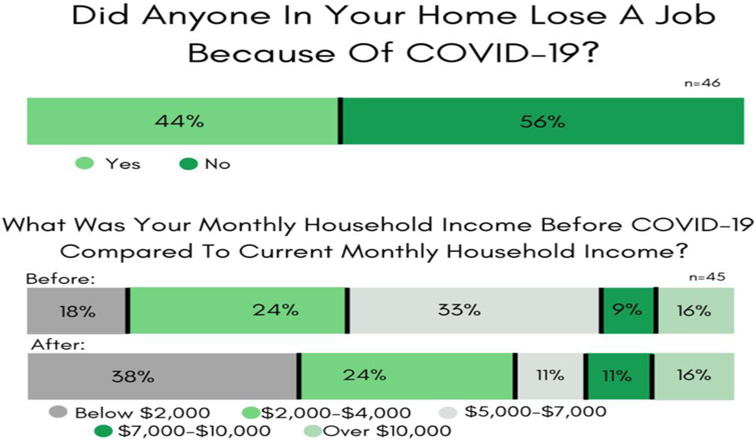
Survey #2 Results. Crisis’s effects on students’ household employment and income. **Source**: Tabulated responses to Survey #2 questions, listed in Appendix 1.

Pre-COVID Figure 2 data shows that 58% (n= 45) of the STEM student households had a >$5,000 monthly income, and the COVID-19 lockdowns resulted in significant decreases in levels of income, as only 38% reported a monthly income above $5,000 (post-COVID). Figure 2 also clearly indicates that the STEM students’ household income losses were not experienced equally.

Survey #2 responses also revealed that Wesley College STEM majors experienced significant personal economic hardship. Before the start of COVID-19, 38% were employed in on-campus positions, and 61% had off-campus jobs. Post-COVID findings indicated that 53% lost their on-campus positions, and 50% got laid off from their off-campus jobs.

The CARES Act (U.S. Department of Education, 2020) established the Higher Education Emergency Relief Fund (HEERF) to provide emergency financial aid grants to students for expenses related to the disruption of campus operations due to COVID-19. Information regarding the CARES Act was posted on the Wesley College website, and all Wesley students received emails about the CARES-HEERF funding program from the office of Wesley’s Dean of Students. Yet on Survey #2, 43% of Wesley’s STEM majors responded that they were clueless about this program. Fourteen STEM majors applied for a CARES-HEERF relief package, and as a result, 43% of the fourteen applicants received CARES-HEERF funding.

#### Survey #3: *COVID-19 effects on STEM students’ resilience*

**Figure-3.**
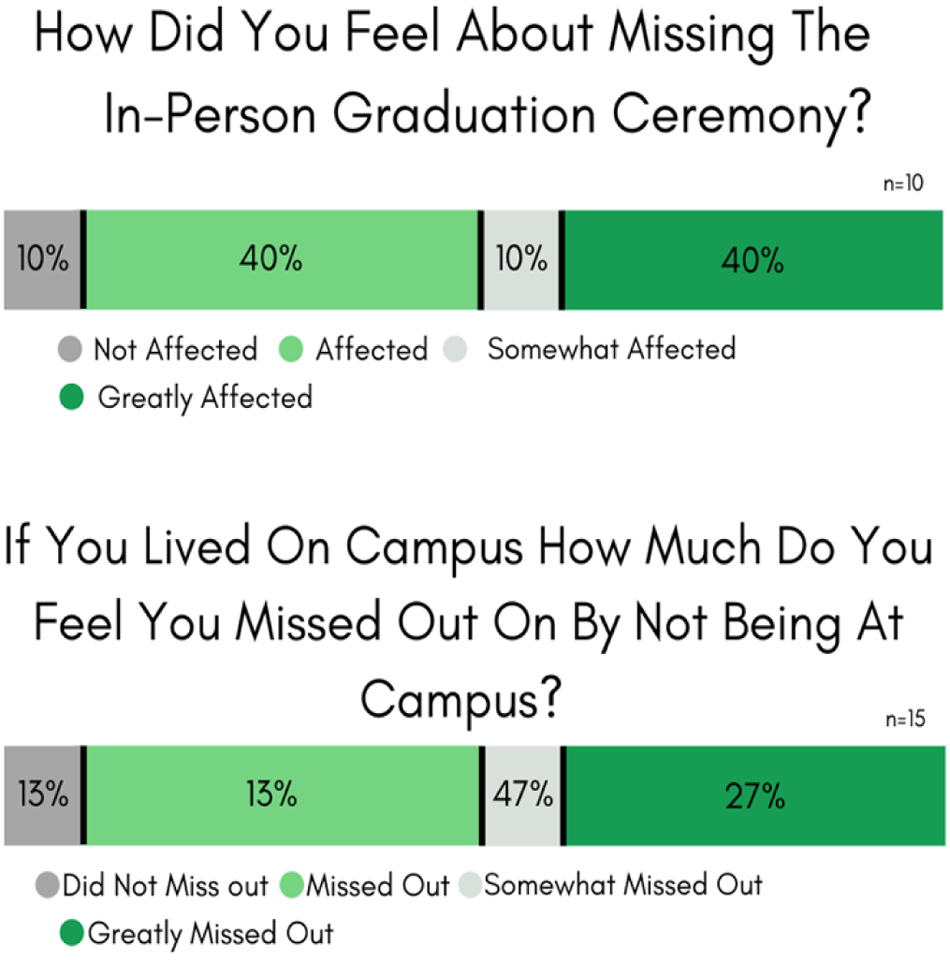
Survey #3 Results. COVID-19 disruptions to graduation and campus life. **Source**: Tabulated responses to Survey #3 questions, listed in Appendix 1.

Parts of Survey #3 included questions that were targeted to graduating seniors and to those nearing graduation. There was apparent frustration about missing out on graduation (see Figure 3). Ninety percent of the graduating Wesley seniors did not like, or favor, the virtual May 2020 graduation format, and 70% indicated a strong preference for a postponed in-person (Fall 2020) ceremony. Furthermore, 80% of the seniors felt that they missed out on peer-to-peer friendships during their final semester, and 73% thought that they did not get a final goodbye with their professors.

Of the 44 Survey #3 STEM student respondents (65% response rate), 9 (20%) indicated involvement in the Spring 2020 athletic programs, and 34% resided on campus. Eighty-seven percent Figure 3 of the on-campus residents missed their campus community during the lockdown (State of Delaware-Delaware Emergency Management Agency, 2020).

Forty-four percent of the STEM majors (n = 44) felt that they missed out on in-person Spring sporting opportunities, and 57% missed their on-campus experience and on-campus learning environment. Additionally, 70% missed formal STEM peer-to-peer mentoring interactions (D’Souza et al., 2018; Neff & D’Souza, 2019) that helped with college success and life, 25% missed the Wesley Science Club activities (Neff & D’Souza, 2019), and 20% missed mentored research presentation opportunities at Wesley’s annual Scholars Day event (D’Souza, 2012; D’Souza et al., 2011; D’Souza et al., 2018; Neff & D’Souza, 2019; Wesley College Grants, 2020).

#### Survey #4: *STEM student opinions about their ability to leverage their skill-sets, and their views about attending in-person classes during the Fall 2020 semester*

**Figure-4.**
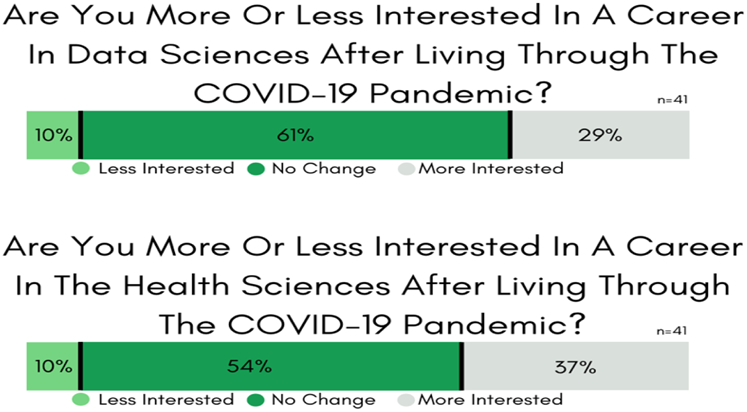
Survey #4 Results. COVID-19 survey indications for reskilling and switching careers. **Source**: Tabulated responses to Survey #4 questions, listed in Appendix 1.

In general, on Survey #4, 41 Wesley STEM majors answered (60% response rate) the majority of the questions. Ten percent of the respondents (see Figure 4) felt that COVID-19 negatively impacted their confidence levels, and these students seriously considered changing majors and their future career goals. On the other hand, in living through the COVID crisis, 29% of STEM majors are more interested in pursuing data science skill-sets, and 37% feel confident in seeking laboratory-health sciences as a career pathway.

Data science and mentored undergraduate research opportunities are available to students as part of Wesley’s liberal-arts core curriculum and through UR-CATS programming (D'Souza et al., 2015; D’Souza, 2012; D’Souza, 2016; D’Souza & Wang, 2012; D’Souza et al., 2015; D’Souza et al., 2011; D’Souza et al., 2015; D’Souza et al., 2017; D’Souza et al., 2018; D’Souza et al., 2018; D’Souza et al., 2014; Fry et al., 2019; Neff & D’Souza, 2019; Wesley College Grants, 2020). Additionally, all majors have access to data science courses (D’Souza et al., 2013; D’Souza et al., 2015; D’Souza et al., 2017; D’Souza et al., 2015; D’Souza et al., 2017; D’Souza et al., 2018; Wesley College Grants, 2020) with collaborative projects and customized platforms for experiments, model training, and testing (D’Souza et al., 2018).

Twelve percent of the Survey #4 STEM program respondents indicated that they were leaving Wesley, and 32% reported that they would seek other higher education solutions if Wesley went with a *remote-only* route in Fall 2020. A majority (60%) indicated a preference for an in-person teaching format even though it meant that they would have to follow stringent mandatory mask-wearing, sanitizing, and social distancing rules.

In mid-August, the College administration disclosed details for de-densified on-campus classes, housing, and dining locations (Clark, 2020; Wesley College, 2020). However, 46% of the STEM majors worried about their ability to pay the 2020-2021 academic year tuition and fees, and 64% are concerned about finding an on- or off-campus job.

#### Survey #5: *COVID-19’s impact on laboratory science courses.*

**Figure-5.**
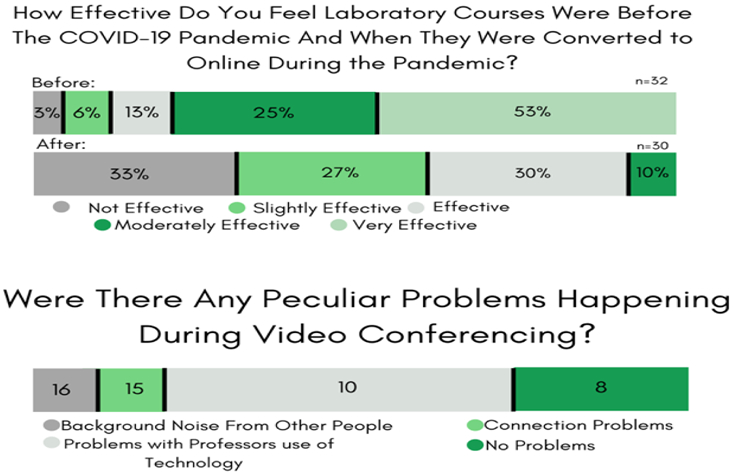
Survey #5 Results. COVID-19 responses to issues relating to laboratory sciences. **Source**: Tabulated responses to Survey #5 questions, listed in Appendix 1.

In Spring 2020, 63% of the STEM students surveyed registered for at least one (hands-on) laboratory science course. Once measures to control the COVID-19 virus were implemented (State of Delaware-Delaware Emergency Management Agency, 2020), their in-lab experience got disrupted, and STEM faculty experimented with electronic means to complete the experiments listed within the course-syllabi. Before transitioning online, 97% of the respondents found the laboratory components of the STEM course to be useful (see Figure 5). This satisfaction with laboratory course effectiveness in the new remote learning format dropped to 67%, and 38% of students were less confident in acquiring (any) new laboratory skills.

Thirty-nine percent of the survey responses indicated a preference for an authentic online laboratory simulation for physical laboratory experiments, over non-video virtual labs or case-studies (22%), and at-home laboratory kits (28%). However, 92% of survey responses reported that STEM faculty were easily accessible outside class hours through email (49%), video conferencing (24%), text messaging (10%), and other apps (5%) or a telephone call (12%). Additionally, in virtual sessions, students reported few interruptions, with the majority interruptions being background noise or connectivity issues.

#### Survey #6 Results. *COVID-19 student responses to their ability to cope during the pandemic*

**Figure-6.**
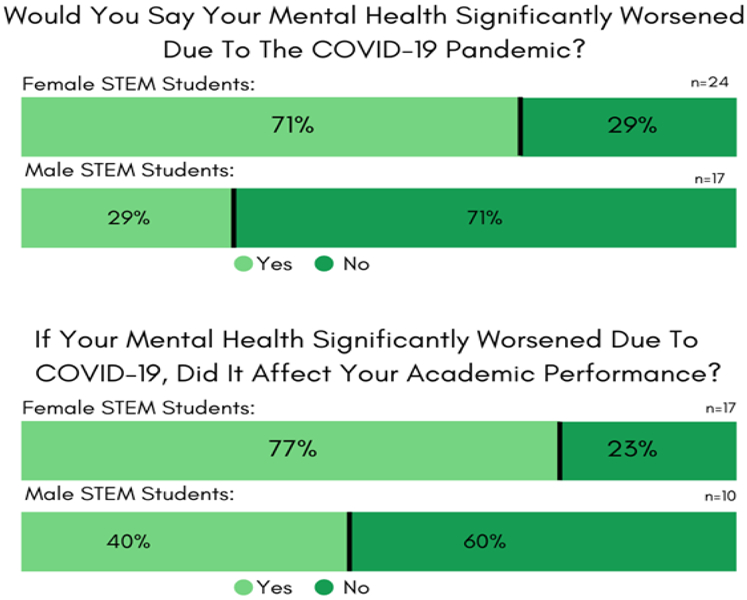
Survey #6 Results. COVID-19 responses regarding motivation and mental health. **Source**: Tabulated responses to Survey #6 questions, listed in Appendix 1.

Sixty percent (41) of the Wesley STEM majors responded to questions relating to their psychological distress due to the COVID-19 crisis. As the lockdown (State of Delaware-Delaware Emergency Management Agency, 2020) days blended together, 66% of the students admitted to having trouble in building and maintaining a daily, as well as a weekly routine. Eighty-nine percent believed that this lack of rituals dinged their Spring 2020 academic work, and only 47% self-reported to any feeling of motivation for study (see Figure 6). Additionally, 48% found it difficult to compartmentalize their (school-work) day at home, 45% saw increased responsibilities in their home environment, and 52% struggled with the online e-learning format.

Delaware’s imposed COVID-lockdown (State of Delaware, 2020) isolation was not exactly conducive to the Wesley STEM majors’ mental health, and 54% of the survey responses expressed negative psychological consequences. Women (71%) were more impacted than men (29%), and 13% of the responses indicated that students felt unsafe about returning home.

Figure 6’s survey data also indicated that the brunt of mental anguish on academic performance is more prevalent in females (77%) than males (40%). Despite the unprecedented challenges, 25% of the students voiced opinions on dysfunctional forms of support from their academic advisors. Still, at the same time, 71% of respondents felt that their professors and advisors showed compassion during the pandemic period, and firmly believed that their professors lowered their expectations about the amount (77%) and quality (66%) of classwork. Overall, 66% of the STEM students reported that they were pleased with their final Spring 2020 grades.

Fear and worry were common mixed emotions revealed in Survey #6 data. Fifty-one percent of the student responses disclosed concerns about their ability to succeed in an online learning environment (63%), STEM degree completion (61%), future career (93%), food (49%), housing (34%), ability to contribute to household income (66%), COVID-19’s impact on student health (85%), COVID-19’s effect on the health of family and friends (93%), caring for family (66%), healthcare access (24%), emotional support (63%), and social isolation (83%).

### 3.2. Tracking of the Wesley STEM Faculty Responses of What College Life is Like during COVID-19

Due to the alarming spike of Northeast COVID-19 cases and the associated COVID-19 mortality numbers, the Delaware Governor issued stay-at-home orders and a coronavirus lockdown in the State (State of Delaware-Delaware Emergency Management Agency, 2020). In response, on Friday, March 13, 2020, the Wesley College President, in consultation with his Coronavirus Action Group (CAG) and his Cabinet (Clark, 2020), decided on a *remote-only* instruction approach that included pandemic-related safety guidance and a request for students to leave campus. The Wesley College administration also issued a directive that beginning Monday, March 16, 2020, Wesley faculty migrate all courses (including the STEM laboratory sciences) to the College’s MyWesley (Jenzebar) platform. Below are the responses received from the Wesley STEM faculty during this disruptive and stressful COVID-19 atmosphere.

#### Faculty Responses: *COVID-19 survey results and interview answers from faculty*

The student co-authors emailed a Google Forms survey to 23 Wesley College STEM faculty (full-time and adjunct) and interviewed 5 through the Microsoft-Teams or the Zoom video conferencing platforms. Nineteen STEM faculty shared survey information (83% response rate) about their metamorphosis in adapting to an online-only instruction model. They also highlighted their mental, physical, social, and economic stressors attributable to the scaling back of campus operations. Among those who provided a written survey response, 58% were female, and 89% were Caucasian.

Traditionally, Wesley STEM faculty experience higher-than-average teaching loads, more service obligations, and more student contact hours to counsel students needing additional support (D'Souza et al., 2015; D’Souza, 2012; D’Souza, 2016; D’Souza & Wang, 2012; D’Souza et al., 2015; D’Souza et al., 2011; D’Souza et al., 2015; D’Souza et al., 2017; D’Souza et al., 2018; D’Souza et al., 2018; D’Souza et al., 2014; Neff & D’Souza, 2019; Wesley College Academic Programs, 2020).

**Figure-7.**
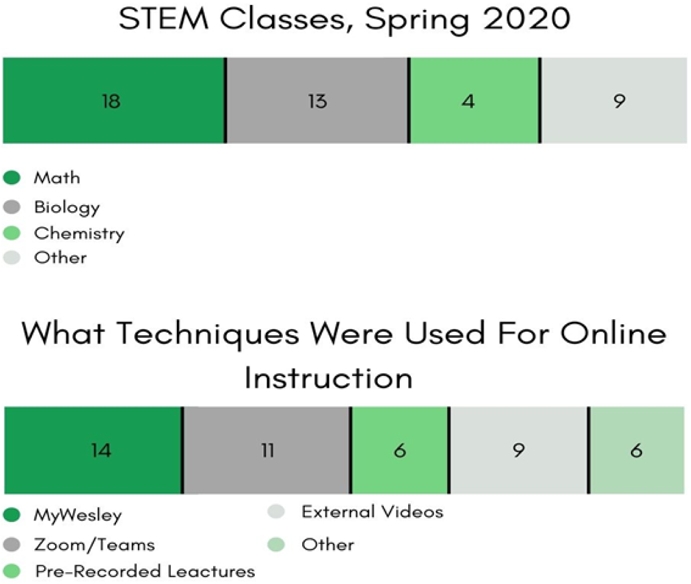
Faculty Survey & Interview Results. Delivery methods for educational content. **Source**: Tabulated responses to Faculty Survey and Interview questions, listed in Appendix 2.

Pre-COVID, 84% of the faculty who responded to the survey had no experience in *any* online mode of instruction. COVID-19 exacerbated their instructional burden of utilizing untested teaching methods in a time of high stress, as they had to (also) attend to marginal students (D’Souza & Wang, 2012; D’Souza et al., 2015; D’Souza et al., 2018). However, in 48 hours, the Wesley STEM faculty quickly transitioned to online hybrid e-learning modes of synchronous and asynchronous forms of interaction and communication (see Figure 7).

Faculty accomplished the video conferencing, teleconferencing, live-chatting, and the live-streaming of lectures using either the Microsoft-Teams or Zoom platforms. For self-guided asynchronous methods of online learning, faculty distributed content (including streaming video content, lecture notes, laboratory assignments, discussion boards, exams, and quizzes) via MyWesley (Jenzebar).

**Figure-8.**
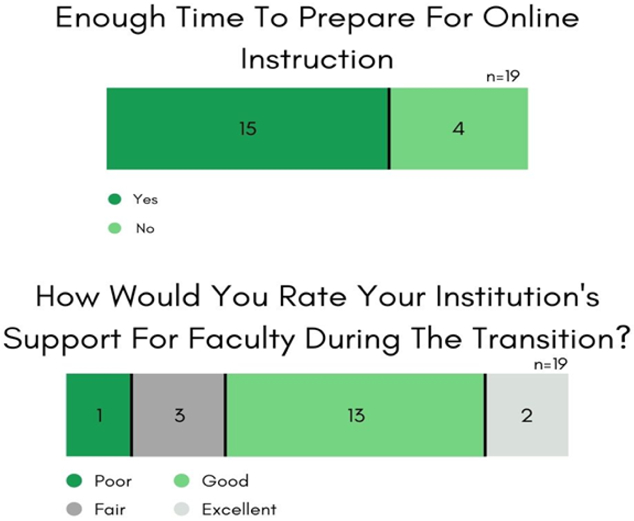
Faculty Survey & Interview Results. Time constraints and institutional support when deploying emergency online learning. **Source**: Tabulated responses to Faculty Survey and Interview questions, listed in Appendix 2.

Some of the STEM faculty respondents found virtual methods of instruction to be impersonal, diminished the quality of their teaching, incompatible with their field of study, and 24% expressed not having enough time to get ready for online classes (see Figure 8). Still, 74% agreed that Wesley and STEM UR-CATS, in particular, had made the appropriate information technology investments to tackle remote instruction, and 79% highly rated the institutional support during COVID.

**Figure-9.**
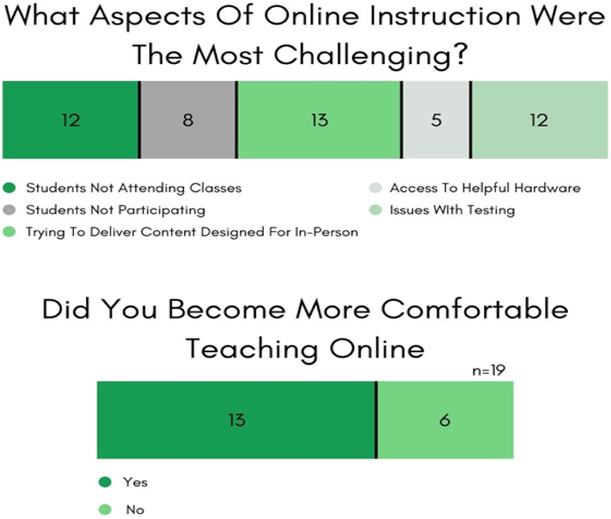
Faculty Survey & Interview Results. Challenges and confidence in online instruction. **Source**: Tabulated responses to Faculty Survey and Interview questions, listed in Appendix 2.

**Figure-10.**
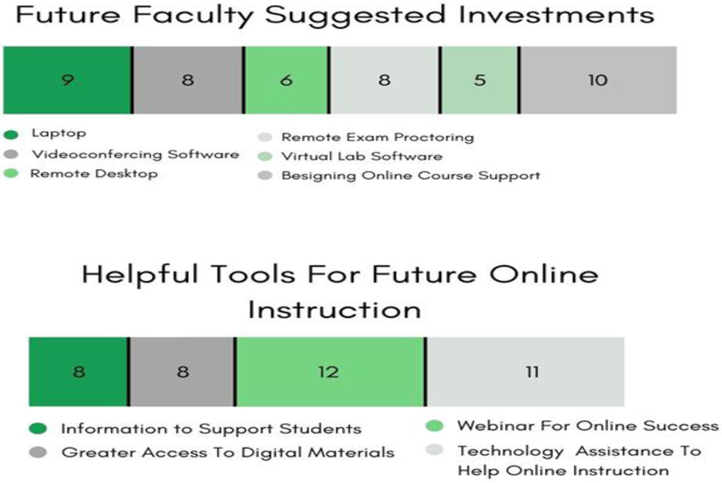
Faculty Survey & Interview Results. Recommendations for Fall 2020. **Source**: Tabulated responses to Faculty Survey and Interview questions, listed in Appendix 2.

Student apathy, classroom engagement, instructional depth (especially for in-person labs and active discussion), and issues with exams (collusion and copying) were common challenges identified by the STEM faculty (see Figure 9). By the end of the Spring 2020 semester, 68% of the Wesley STEM faculty felt adaptable enough and expressed complete confidence in a remote e-learning environment. For training and resources, the Wesley STEM Faculty also made some recommendations (see Figure 10) to coalesce around various functional areas, and that ease the shift to digital learning further.

**Figure-11.**
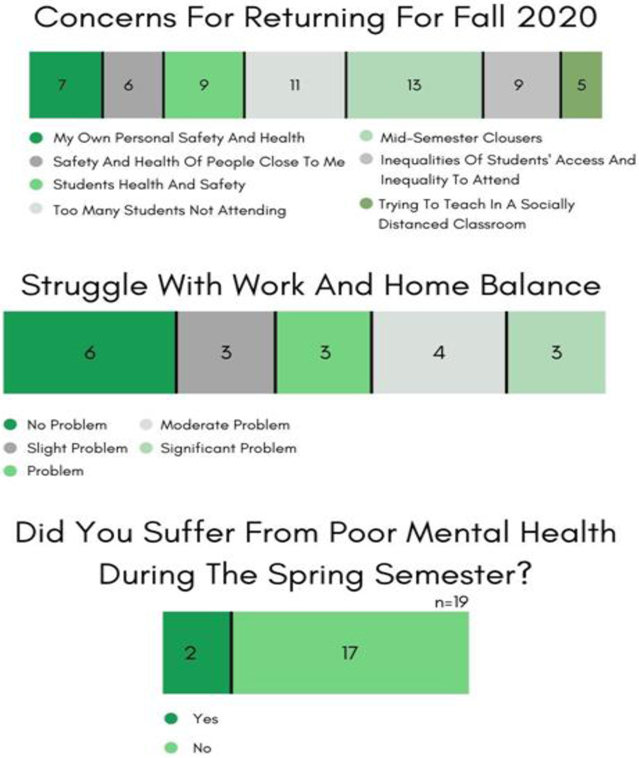
Faculty Survey & Interview Results. Looking ahead and tackling the COVID crisis. **Source**: Tabulated responses to Faculty Survey and Interview questions, listed in Appendix 2.

The extended prevalence of the pandemic has affected the family, mental and psychological states of the Wesley STEM Faculty (see Figure 11). Thirty-seven percent of the faculty felt that the *remote-only* e-learning mode created chaos and a stressful atmosphere with their work-family balance, and 11% admitted to a much lowered mental health level. This mental health stress bubbles quietly into the projected Wesley STEM faculty concerns (see Figure 11) for the far-from-ideal living and learning environments that the College has actively designed for Fall (Clark, 2020; Wesley College, 2020).

## 4. Conclusions

Once the Wesley College administration decided to move to virtual instruction midway through the Spring 2020 semester (March 2020), then within the 48 weekend hours, the Wesley STEM faculty confronted the challenge of transitioning both lectures and labs to synchronous and asynchronous e-learning states. During the COVID-19 lockdown, the STEM majors encountered stumbling blocks in their economic dislocation, home and social life, equity of learning opportunities, food insecurities, and mental health concerns. Yet, due to STEM faculty mobilizing to support digital e-learning, Wesley student adaptability, and the seamless transition into what was happening in STEM coursework pre-COVID, 66% of the students reported that they were pleased with their final Spring 2020 grades.

## Student Survey Questions - Appendix 1

Questions asked in all six student surveys: What is Your Race? And What is Your Gender?

### Survey #1: Technology

1. Do you have Wi-Fi at home to access online work?
2. If no, how hindered do you feel by the lack of internet access?
3. Do you have access to a printer/scanner in your home?
4. If no, did you feel hindered by the inability to print/scan in your home?
5. Do you have a laptop?
6. If no, did Wesley provide a laptop?
7. What platforms did your professors use?
8. Did you have trouble learning how to access the online platform?
9. If yes, how much trouble did you experience learning how to access the online platform?
10. How long did it take you to adjust to this new learning style?

### Survey #2: House-Hold Income

1. What was your monthly household income before the COVID-19 pandemic?
2. What was your monthly household income during the COVID-19 pandemic (now)?
3. Were you employed on-campus at Wesley College before COVID-19?
4. If yes, did the school find ways for you to work remotely? (Explain)
5. Were you employed outside of campus before COVID-19?
6. If yes, did you lose your job as a result of COVID-19?
7. Did anyone in your home lose a job because of COVID-19?
8. If yes, how much do you believe this affected your learning?
9. Were you aware of the available aid under the CARES Act from the Higher Education Emergency Relief Fund?
10. Did you apply for the available aid under the CARES Act from the Higher Education Emergency Relief Fund?
11. If you applied, did you receive the available aid under the CARES Act from the Higher Education Emergency Relief Fund?
12. Is there anything related to the topic of income or technology that was not covered, you feel we should know?

### Survey #3: Residential Community (broken-up by seniors and underclassmen)

1. Questions for Both:
  1. Are you a student-athlete?
  2. If you are a student-athlete, how much do you feel you missed out on your season due to COVID-19 this Spring Semester (including tournaments, games, events, etc.)?
  3. Did you live on campus?
  4. If yes, how much do you feel you missed out by not having the residential community environment at Wesley, due to the COVID-19 pandemic?
  5. Did you enjoy being off campus (at home) more than being on campus concerning ‘college life.’
    - Please follow up with any comments you’d like to make about the above question?
  6. If yes, how much do you feel you missed out by not having the residential community environment at Wesley, due to the COVID-19 pandemic?
  7. What Wesley College events were you most looking forward to?
  8. Is there anything else related to the topic of community and COVID-19 you think we should know?
2. Senior Questions:
  1. How much do you feel missing out on graduation has affected you?
  2. Were you happy with the online graduation format?
  3. Would you have instead not had an online ceremony and preferred to wait for an in-person opportunity?
  4. Are you interested in attending a postponed graduation ceremony?
  5. If you would be interested in attending a postponed graduation ceremony, would you be more available in the Fall or Spring?
  6. How much do you feel not getting to say goodbye to your peers and professors have affected you?
3. Underclassmen Questions:
  - How much do you feel like you missed out on peer mentorship?

### Survey #4: Career Interests and Occupational Choices

1. Are you planning on returning to Wesley College in the Fall of 2020?
2. If not returning, please explain why.
3. As a result of COVID-19, did you change your major?
4. If yes, what was your major before, and what is now your new major?
5. If you did change your major due to COVID-19, what was the reasoning?
6. If you have not changed your major due to COVID-19, are you thinking of changing your major due to COVID-19?
7. If you are thinking of changing your major due to COVID-19, what is the reasoning?
8. How confident are you in the next steps you need to take to stay on track amid the outbreak?
9. Are you more or less interested in a career in the health sciences after living through the COVID-19 pandemic?
10. Due to COVID-19, were your career interests changed or affected?
11. If your career choices were affected by COVID-19, please explain why and what changed?
12. Would having the Fall of 2020 semester online at Wesley cause you to do any of the following?
13. If students were to return on campus in the Fall of 2020 semester and had to follow social distancing and mask-wearing, how happy would you be about returning?
14. Would you rather have to follow social distancing and mask rules and come to campus for the Fall of 2020 semester, or would you rather stay home like the end of the spring 2020 semester?
15. How much more or less concerned are you about paying for the next year of college as a result of COVID-19?
16. How worried are you about finding a job following the COVID-19 pandemic?
17. Is there anything else we should know related to the topic of COVID-19 and career interests or returning to campus that we did not cover in this survey?

### Survey #5: Education

1. Were you taking a lab course during the Spring of 2020 Semester?
2. If you were taking a lab course during the Spring of 2020 semester, what was that course? (If you took multiple lab courses, please list all of them)
3. If you were in a lab course, which courses did not provide lab simulations to supplement the missed education during labs?
4. If you were in a lab course that provided an online stimulation, which type of method was used?
5. In your opinion, how could you and your peers gain necessary lab skills while still learning science better and more efficiently in a virtual environment?
6. How effective do you feel laboratory courses were during the first half of the Spring 2020 Semester (before the transition to online)?
7. How effective do you feel laboratory courses were once converted to online during the COVID-19 pandemic?
8. Do you feel more or less confident in your laboratory skills after experiencing online labs?
9. Were there any particular problems happening during video conferencing? (Check all that apply or feel free to elaborate more in the other option)
10. If you did have peculiar problems happening during video conferencing, how did it affect you, and what could the College have done to help?
11. Was faculty accessible outside of class time?
12. If faculty was accessible outside of class, how did your professors format their office hours for questions outside of your classes?
13. Is there anything else we should know related to the topic of COVID-19 and the issue of education about lab courses and online formatting?

### Survey #6: Mental Health

1. Were you happy with your academic performance in the Spring of 2020?
2. If no, what in particular do you think prohibited your performance? (What stopped you from doing better?)
3. How well would you rate your motivation before COVID-19?
4. How motivated were you during online learning?
5. If you struggled with motivation, what factors contributed to this?
6. Did you have trouble maintaining a routine during COVID-19?
7. If you did have trouble maintaining a routine, please rate how much it affected your performance
8. How well would you rate your mental health during online learning?
9. Would you say that your mental health significantly worsened due to the COVID-19 pandemic?
10. If yes, did your mental health affect your academic performance?
11. How would you compare the following to before the COVID-19 pandemic? [Health Care Access]
12. How would you compare the following to before the COVID-19 pandemic? Ability to pursue your studies (including graduation and degree completion)]
13. How would you compare the following to before the COVID-19 pandemic? [Ability to Socialize]
14. How would you compare the following to before the COVID-19 pandemic? [Overall psychological wellbeing, including feelings of anxiety or depression]
15. To what extent are you concerned about the following? Access to consistent education]
16. To what extent are you concerned about the following? Access to consistent food]
17. To what extent are you concerned about the following? Access to consistent housing]
18. To what extent are you concerned about the following? [Caring for family members financially]
19. To what extent are you concerned about the following? [Financial impact on my life]
20. To what extent are you concerned about the following? [How COVID-19 will impact my health]
21. To what extent are you concerned about the following? [How COVID-19 will impact the health of my family and friends]
22. To what extent are you concerned about the following? [How this situation will impact my academic future]
23. To what extent are you concerned about the following? Lack of emotional support]
24. To what extent are you concerned about the following? [My ability to succeed in an online academic environment]
25. To what extent are you concerned about the following? [Social isolation]
26. Did you feel physically and emotionally safe returning to your home during the COVID-19 transition?
27. Would you feel emotionally and physically safe if you had to return to campus this Fall? (Ex: Are you scared of going back into the public and catching the virus from campus?)
28. Have you had an increase in family responsibility (chores, providing online learning for your siblings/family members, etc.)?
29. If yes, how much has this affected your academic performance?
30. Have you found it challenging to balance family life and school-work?
31. How much do you agree with the following statement, “I feel supported by my professors and advisors”?
32. Do you feel like your professors and advisors were compassionate and understanding during the pandemic?
33. Do you feel comfortable talking to your professors/advisors about your life challenges?
34. How much do you agree with the statement that your professors and faculty had lowered their expectations about the amount of work you could do?
35. How much do you agree with the statement that your professors and faculty had lowered their expectations about the quality of work you could do?
36. Which of the following online or virtual resources would you consider utilizing if Wesley offered them?
37. Which ways did COVID-19 most impact you?
38. What self-care practices have been most challenging?
39. Compared to before COVID-19, how do you feel in general about: [The Next Month]
40. Compared to before COVID-19, how do you feel in general about: [Your Summer Plans]
41. Compared to before COVID-19, how do you feel in general about: [Your longer-term plans]
42. What ONE word describes how you feel about the campus closing due to the COVID-19 outbreak?
43. In your opinion, what could Wesley College have done to provide better mental health resources?

## Faculty Questions – Appendix 2

### Faculty Survey Questions

1. What is your race?
2. What Is your gender?
3. What courses were you responsible for teaching during the spring semester of 2020?
4. Have you ever taught an online course at Wesley College?
5. If you have taught an online course before the Spring of 2020 semester, please elaborate on how the course was set up (ex. Platforms used, structure, format, grading).
6. What teaching techniques did you use in the Spring of 2020?
7. Did you lower your expectations about the amount of work your students were able to do?
8. Did you lower your expectations about the quality of work your students were able to do?
9. Did you drop any course requirements that were initially planned before the COVID-19 pandemic? (ex. exams, assignments, readings)
10. How much do you agree with the following statement, “I changed the kinds of assignments or exams I asked my students to complete”?
11. Overall, how would you rate your institution’s support for faculty and professors in transitioning to remote teaching in the Spring of 2020 semester?
12. What could have been done to better support faculty and professors during this transition?
13. If you could change one thing about your experience with remote teaching, what would it be?
14. For online teaching: which aspects of online education were most challenging for you? (Check all that apply)
15. For the online teaching during the Spring of 2020 semester, what did you find most successful, please describe?
16. Take a minute to think back to the first week of remote teaching. Comparing how you feel now to how you felt then, how has your experience of teaching in a remote environment changed?
17. How much do you agree with the following statement, “my institution has made previous investments in information technology that positioned us well to respond to the COVID-19 pandemic”?
18. How much of a problem did you have balancing work with home life? (ex: online schooling your children, domestic chores, etc.)
19. Did you, or anyone else in your family contract COVID-19?
20. If yes, please explain the impact it had on your family and the implications on your work.
21. How would you rate your mental health while teaching during the online Spring 2020 semester due to the COVID-19 pandemic?

### Faculty Interview Questions

1. What did you teach?
  - How was it taught? Please elaborate on how you set up your class
2. Were you pleased with your ability to transition into the online format?
3. What do you think Wesley College could have done differently at the beginning of the pandemic? (Before being shut down and when we shut down)
4. Were you independently preparing for online classes before the official shut down (spring break week)?
5. How much communication did you have with the administration before the decision to move online?
  - Could there have been better communication, and if so, what would you have wished to happen?
6. Do you have kids? Age?
  - Did you split childcare with your partner?
  - How did you manage to parent and to work from home?
  - Was this a struggle with you?
7. Did you struggle with time management? (Avoiding distractions like the TV, cellphones, etc.)
8. If you lowered your expectations, please explain your reasoning.
9. If you did not lower your expectations, explain your reasoning.
10. Did you change the assignments you required?
  - If you did, explain your reasoning
  - If you did not, explain your reasoning
    - If you did not, do you wish you would have?
11. Were you happy with your students?
  - Participation?
  - Grades?
  - Other?
12. Did you have adequate access to technology?
  - What would you have wanted Wesley to provide?
13. In the Fall, how are you going to adapt your courses?
  - Do you have a plan for in-person?
  - Do you have a plan for online? (is Wesley encouraging/requiring this?)
14. What training would you like to receive from Wesley in the next semester?
15. In your opinion, what do you think is the best way to deal with labs?
  - What do you think would be the best way to continue labs online if we will need to?
16. What is your biggest concern?
17. Did anything surprise you about your students this past semester?
18. What was the biggest frustration you had throughout this entire process?
19. Do you think your responsibilities as an educator change when teaching an online class as opposed to a traditional college course?
20. What questions/concerns do you have about teaching in a socially distanced classroom?
  - Student access to mask?
  - Students' willingness to wear a mask?
  - Ability to concentrate with a mask on?
  - What types of masks would you prefer, would you want face-shields instead for teaching?
  - Other?
21. Are you worried about academic dishonesty during online classes, and if we need to go online next semester, how do you think it’s best to handle this issue?
  - Do you think making personal assignments and different version tests will work, or are there other things you have in mind?
  - Could Wesley College somehow help with this issue, and if so, how?
22. Did communication between your department suffer?
  - Would you have benefited from frequent department meetings?
23. In your opinion, why do you feel like students succeeded or did not succeed?
  - Laziness?
  - Mental Health?
  - Job?
  - Other?
  - Student attitude/perseverance?
24. Overall, how much stress did COVID have on you?

How connected did you feel to Wesley College from March through June?
